# Ultrasound and Radiation-Induced Catalytic Oxidation of 1-Phenylethanol to Acetophenone with Iron-Containing Particulate Catalysts

**DOI:** 10.3390/molecules25030740

**Published:** 2020-02-08

**Authors:** Mohamed M. A. Soliman, Maximilian N. Kopylovich, Elisabete C. B. A. Alegria, Ana P. C. Ribeiro, Ana M. Ferraria, Ana M. Botelho do Rego, Luís M. M. Correia, Marta S. Saraiva, Armando J. L. Pombeiro

**Affiliations:** 1 Centro de Química Estrutural, Complexo I, Instituto Superior Técnico, Universidade de Lisboa, 1049-001 Lisboa, Portugal; mohamed.soliman@tecnico.ulisboa.pt (M.M.A.S.); pombeiro@tecnico.ulisboa.pt (A.J.L.P.); 2 Centro de Química e Bioquímica, Departamento de Química e Bioquímica, Faculdade de Ciências, Universidade de Lisboa, 1749-016 Lisboa, Portugal; mssaraiva@fc.ul.pt; 3 Área Departamental de Engenharia Química, ISEL, Instituto Politécnico de Lisboa, 1959-007 Lisboa, Portugal; luiscorreia1994@gmail.com; 4 BSIRG, IBB-Institute for Bioengineering and Biosciences, Departamento de Engenharia Química, Instituto Superior Técnico, Universidade de Lisboa, 1049-001 Lisboa, Portugal; ana.ferraria@tecnico.ulisboa.pt (A.M.F.); amrego@tecnico.ulisboa.pt (A.M.B.d.R.); 5 BioISI-Biosystems & Integrative Sciences Institute, Departamento de Química e Bioquímica, Faculdade de Ciências, Universidade de Lisboa, Campo Grande, 1049-001 Lisboa, Portugal

**Keywords:** iron catalysts, alcohol oxidation, ball-milling, mechanochemical synthesis, microwave irradiation, ultrasound, induction heating, magnetic recovery

## Abstract

Iron-containing particulate catalysts of 0.1–1 µm size were prepared by wet and ball-milling procedures from common salts and characterized by FTIR, TGA, UV-Vis, PXRD, FEG-SEM, and XPS analyses. It was found that when the wet method was used, semi-spherical magnetic nanoparticles were formed, whereas the mechanochemical method resulted in the formation of nonmagnetic microscale needles and rectangles. Catalytic activity of the prepared materials in the oxidation of 1-phenylethanol to acetophenone was assessed under conventional heating, microwave (MW) irradiation, ultrasound (US), and oscillating magnetic field of high frequency (induction heating). In general, the catalysts obtained by wet methods exhibit lower activities, whereas the materials prepared by ball milling afford better acetophenone yields (up to 83%). A significant increase in yield (up to 4 times) was observed under the induction heating if compared to conventional heating. The study demonstrated that MW, US irradiations, and induction heating may have great potential as alternative ways to activate the catalytic system for alcohol oxidation. The possibility of the synthesized material to be magnetically recoverable has been also verified.

## 1. Introduction

Alcohols and related compounds, e.g., diols or benzoins, are among the most abundant and used starting synthetic materials, and their partial oxidation can provide a significant added value to a number of chemical processes, since many aldehydes, ketones, and carboxylic acids are important by themselves and also can be used for further processing [1,2,3]. Generally, alcohols are relatively cheap and non-toxic raw materials, and can be easily stored, transported, and dissolved [1,2,3,4]. Moreover, alcohols can be produced from renewable resources such as starches, molasses, grains, etc., [2,5]. As a result, it was estimated that the global industrial alcohol market was about USD 106 billion in 2016 [6].

Many transition-based catalysts are currently used for the mild partial alcohol oxidation [7], and non-toxic iron is one of the most attractive components owing to several reasons [8,9,10,11]. Apart from the recognized catalytic activity in many oxidation processes, magnetic properties of the iron-containing particles can lead to beneficial features, such as the possibility to activate a catalyst with an oscillating magnetic field of high frequency (so-named induction heating) or to recuperate it with a permanent one [9,12]. However, solution-based “wet” preparations of the iron-containing materials is not a trivial task because of the strong hydrolyzing ability of iron salts. The iron hydrolysis and the subsequent iron hydroxide precipitation significantly complicate preparative procedures and make them difficult to achieve. In contrast, solvent-free “dry” mechanochemical processes allow preparing iron-containing and other related materials simply and directly [9,10,13]. Additionally, not much is known about how iron-containing catalytic materials would respond to inductive heating or mechanical stimuli, e.g., to ultrasound [11,14,15]. Thus, it would be attractive to find ways for the straightforward synthesis of particulate iron-containing materials and to study their catalytic behavior under various energy inputs, such as microwave, ultrasound, and oscillating magnetic irradiations.

Therefore, in this work, we performed the preparation of new iron-containing dispersed materials by simple one-pot wet, dry, and combined methods, and studied their activity and reusability in the oxidation of secondary alcohols to the corresponding carbonyl-containing compounds under various energy inputs. 1-Phenylethanol was selected as a model substrate, while the conventional and induction heating, MW- and US-irradiations were used to activate the proposed catalytic system.

## 2. Results and Discussion

### 2.1. Characterization of the Dispersed Materials

The catalytic materials were prepared by “wet” (solvent-based) for **1**, “dry” (ball-milling) for **3** and **4**, and combined (for **2**) methods as described in Experimental section and Supplementary Materials (Figure S1, Table S1). Their characterization was performed by Fourier-transform infrared (FTIR), UV-Vis diffuse reflectance (DRS), energy-dispersive X-ray (EDX) spectroscopies, powder X-ray diffraction (PXRD), thermogravimetric analysis, scanning electron (SEM), and field emission gun scanning electron (FEGSEM) microscopies. 

The FTIR analysis of the prepared materials (Figures S2 and S3) revealed the presence of O-H moieties for **1** and **2** (peaks in the 3300–3400 and 1600–1700 cm^−1^ ranges), while peaks at 500–600 cm^−1^ can be attributed to the stretching vibration of the Fe-O bond [16]. In **3** and **4**, the peaks at ca. 1400 cm^−1^ can be associated to iron oxyhydroxide [17], while the bands at 1229, 1140, 998, and 626 cm^−1^ are related to the S=O group [18,19]. These results relate well with the data obtained by thermogravimetric analysis (Figure S4), where elimination of the coordinated water and hydroxy groups can be observed at 100–300 °C while the S=O group decomposition occurs at 500–700 °C. 

The DRS spectra (Figure S5) are presented in terms of *F*(*R*), estimated from the recorded reflectance (*R*). The absorption bands of **3** appear at 255 and 282 nm whereas the absorption for **4** (541 nm calculated from the deduced band gap energy following the Kubelka–Munk spectra) [20] falls in the visible region. The variation in the estimated adsorption and the band energy gap indicates that the sample optical behavior is determined by the addition of NaOH to **3** to give **4**.

**Figure 1:**
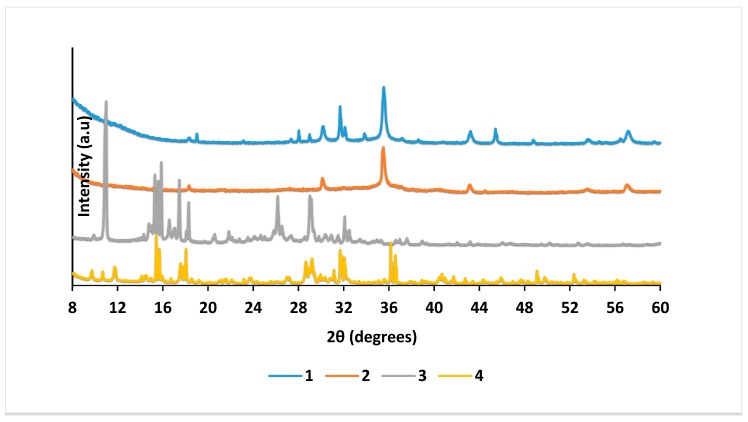
Powder X-ray diffraction (PXRD) patterns of **1**–**4**.

The PXRD patterns of **1** and **2** (Figure 1 and Figure S6) are quite alike, and both demonstrate the presence of iron oxide magnetite. They do not exhibit hematite peaks, but PXRD of **1** displays the occurrence of ferrimagnetic iron oxide maghemite (γ-Fe_2_O_3_) with the same spinel ferrite structure as magnetite [21]. It can be assumed that both **1** and **2** contain a mixture of maghemite or magnetite, although in **2** the dominant phase is magnetite. The magnetic susceptibility measurements (Table S2) relate well with the obtained PXRD results.

The PXRD spectra of **3** and **4** (Figure 1, Figure S6 and S7) reveal that most of the peaks correspond to the ferric oxyhydroxide FeO(OH), akaganeite Fe^+3^O(OH) with some iron oxide hydroxide goethite Fe^+3^O(OH) and cubic magnetite Fe_3_O_4_ as traces [21]. The oxyhydroxide prepared from ferric chloride is usually the β polymorph (akaganeite), often in the form of thin needles [21]. 

**Figure 2:**
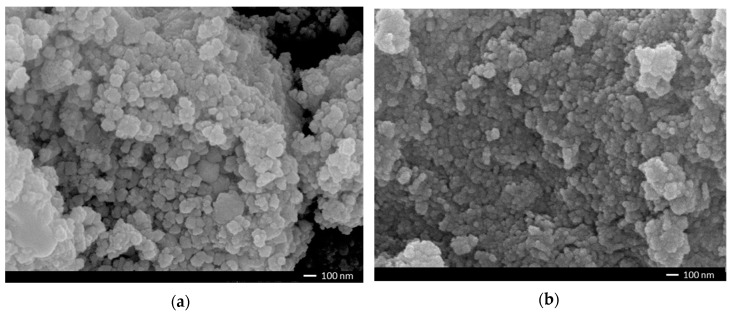

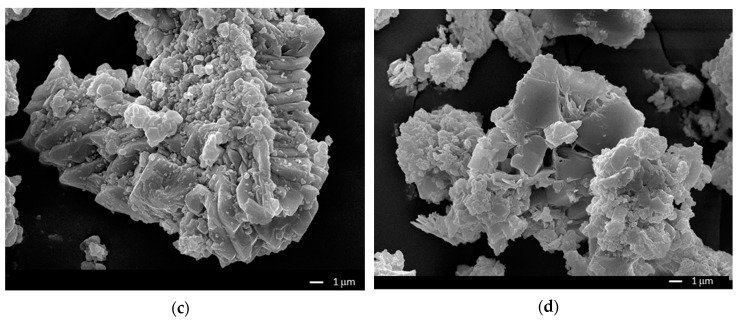
Scanning electron microscopy (SEM) images of **1** (**a**), **2** (**b**), **3** (**c**) and **4** (**d**).

According to the performed SEM imaging, the prepared materials possess distinctive morphologies: spherical particles are observed for **1** and **2** (Figure 2a,b) and needles and rectangles for **3** and **4** (Figure 2c,d). The needle like morphology may be due to the formation of goethite α-FeOOH [22].

**Figure 3:**
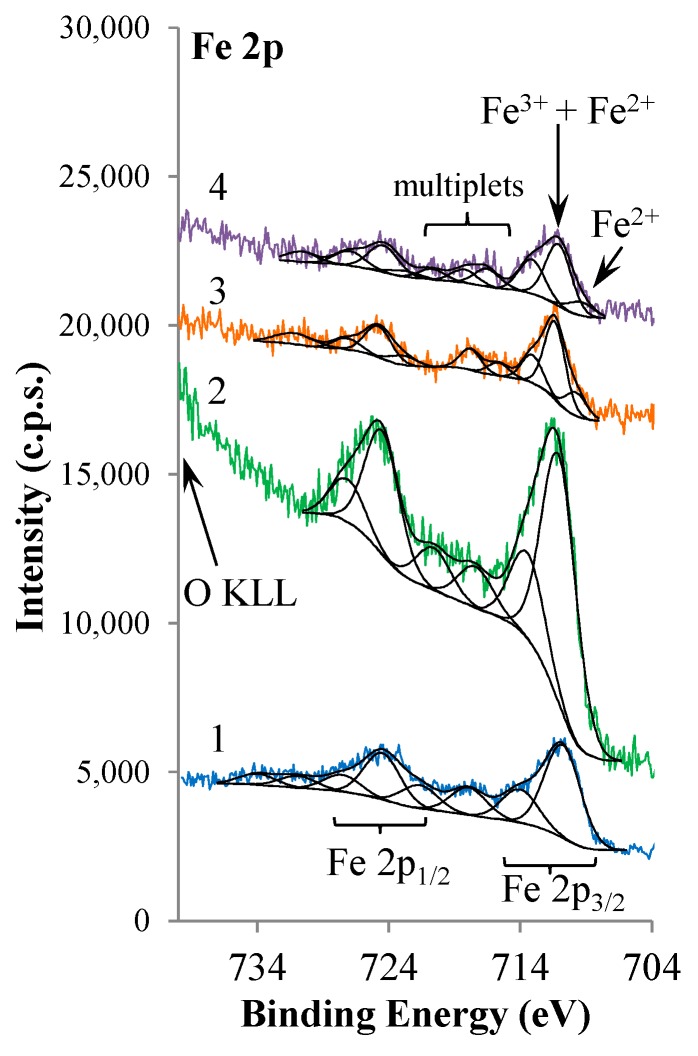
Fe 2p X-ray photoelectron spectroscopy (XPS) regions of **1**–**4**.

For the XPS studies of samples **1** and **2**, the charge shifts were corrected using as reference the binding energy (BE) of aliphatic carbon, centered at 285 eV. The use of such BE reference for samples **3** and **4** was not enough to shift Fe 2p regions to sound corrected positions; the main peak being around 712 eV, which is slightly larger than the reported BE for Fe^3+^ [21]. In Figure 3, Fe 2p regions for samples 3 and 4 are plotted using a different charge correction, adopted to reach a compromise between the information taken from the quantification (Table 1), the qualitative analysis of spectra and previous results obtained for different iron species [23]. For these samples, the BE of the carbonaceous graphitized contamination was set to 284.4 eV, which seems to yield coherent results for all the other elements. Moreover, the samples, where the reference BE was needed to be 284.4 eV, show a much lower charge accumulation. The two facts are compatible with each other since an overlayer of graphite on the samples would increase the electron conduction at the surface. It seems that a longer ball milling process promotes the graphitization of the carbon contaminations.

Both Fe 2p regions of **1** and **2** look like that of Fe_3_O_4_ analyzed previously [23], with Fe 2p_3/2_ components being centered at 710.9 ± 0.2 eV. The overall quantification (Table 1) shows that almost no Cl, N, and S remain, which is compatible with the reaction of the iron precursors and consequent production of the iron oxide, mainly Fe_3_O_4_ with some traces of the Fe(II) salt. Two main differences exist between **1** and **2**; the presence of a relatively high quantity of Na in **1**, which is absent in **2**; and a larger amount of carbonaceous species in **2**. It is worth noting that the ratio O_530 eV_/Fe between the relative amount of oxygen bound to iron (found at 530.0 ± 0.1 eV) and iron in **1** is the same (within the experimental error) as the nominal ratio in Fe_3_O_4_ (1.33). In **2**, this ratio is larger, because the relative amount of iron is underestimated because of the intense O KLL Auger peak around 743 eV (for the spectra acquired with Mg Kα). This outline is an overlapping of multiple peaks at the high BE side of Fe 2p_1/2_, which cannot be quantified in this case.

The profiles of **3** and **4** are closer to Fe(II) species, as attested by the Fe(II) spectrum analyzed previously [23]. However, significant amounts of Cl (BE ~198.7 eV) remain in samples, particularly in **3** with a Cl/Fe atomic ratio of 2.7, which is close to the nominal one in FeCl_3_. Additionally, the nitrogen amount is relatively high in both **3** and **4** (BE between 400.7 and 402.2 eV), and some sulphur from sulphate groups (BE ~169 eV) is detected. These results led to the assignments indicated in Figure 3; while in **1** and **2** the main peaks are from the mixed Fe(II, III) oxide (Fe_3_O_4_), in **3** and **4**, the main peak is attributed to Fe^3+^ from the FeCl_3_ precursor mixed with some Fe^2+^ from the other precursor.

### 2.2. Catalytic Studies

#### 2.2.1. Catalysts Screening

**Scheme 1:**
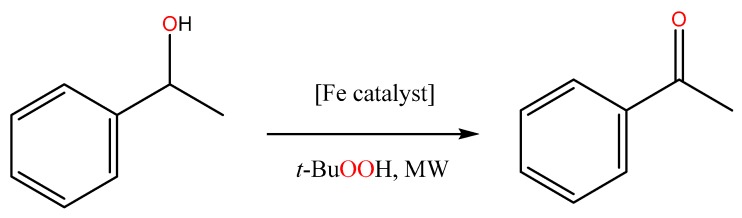
Solvent-free oxidation of 1-phenylethanol to acetophenone.

The prepared materials **1**–**4** and, for comparative purposes, the starting iron salts FeCl_3_·6H_2_O, FeSO_4_·(NH_4_)_2_(SO_4_)·6H_2_O, and anhydrous FeCl_3_ were screened for the microwave-assisted oxidation of 1-phenylethanol using *tert*-butyl hydroperoxide (1 eq. of *t*-BuOOH, aq. 70%) as oxidant. We choose the mentioned model reaction and particular experimental conditions (Scheme 1, Table 2) because of our longstanding interest in such type of reactions and previous experience [2,4,9,12,13,14,15,24,25]. Blank experiment (in the absence of any catalyst), was undertaken and no conversion was observed (Table 2, entry 1). Additionally, the effect of the catalyst amount, ranging from 0.05 to 5 mmol, was also studied (Table 2). Among the studied materials, the catalysts **1** and **2**, obtained by the wet and combined methods, exhibit the lowest activities (yields up to ca. 3 and 11% for **1** and **2**, respectively), whereas the catalysts **3** and **4** obtained by ball milling perform better with acetophenone yields of 58 and 65%, respectively (Table 2, entries 15 and 19). 

The modest performance of **1** and **2** can be related to the presence of magnetite (Table S2), which favors agglomeration of particles, and consequently the surface reduction. Besides, lower solubility can also play a negative role. In contrast, in **3** and **4**, the mechanochemical treatment promotes the iron hydroxide and oxyhydroxide formation, which were shown to be active catalysts for various oxidation reactions [26].

#### 2.2.2. Optimization of Parameters

**Figure 4:**
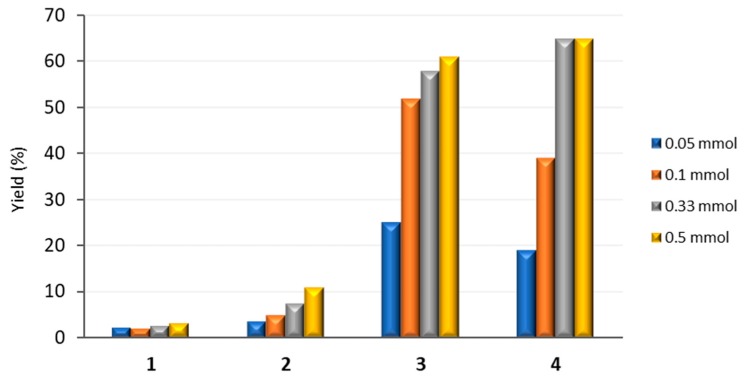
Effect of the catalyst amount (0.05–5 mmol) on acetophenone yield (mol%, vs. substrate) in the MW-assisted peroxidative oxidation of 1-phenylethanol, catalyzed by **1**–**4**. Reaction conditions: 5 mmol substrate, 5 mmol *t*-BuOOH (aq. 70%), 80 °C, 1 h, MW irradiation (5–10 W).

As it can be expected, the yields grow with increase of the catalyst amount, e.g., from 25% and 19% to 58% and 65% upon changing the amount of catalyst from 0.05 mmol to 0.33 mmol (Figure 4, Table 2, entries 13–16, for **3**, and 17–20, for **4**, respectively). However, beyond 0.33 mmol of catalyst, the yields do not grow substantially. Thus, the reaction parameters for the most active catalysts **3** and **4** were further optimized considering this amount of catalyst (0.33 mmol).

**Figure 5:**
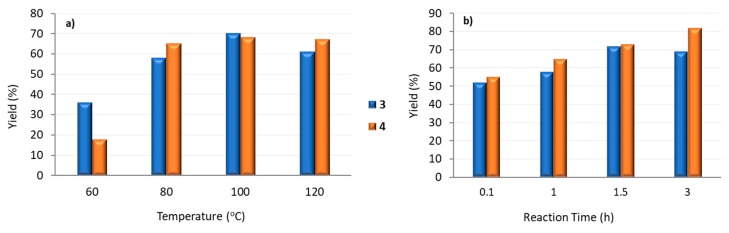
Effect of temperature (**a**) and reaction time (**b**) on acetophenone yield in the MW-assisted peroxidative oxidation of 1-phenylethanol, catalyzed by **3** and **4**. Reaction conditions: 5 mmol substrate, 5 mmol *t*-BuOOH (aq. 70%), 60–120 °C, 0.1–3 h, MW irradiation (5–10 W).

The temperature effect for catalysts **3** and **4** (Table 3, Figure 5a) was studied at 60, 80, 100, and 120 °C, and 80 °C was found to be the best option since the use of higher temperatures (100 and 120 °C) did not result in a significant increase in the product yield. The reaction was also carried out at different times (0.5–3 h), and it was demonstrated that more than half of the alcohol had been converted to ketone after a period of 0.5 h (Table 3, entries 1-4 and entries 13-16, for **3** and **4**, respectively). After 1.5 h, more than 70% of 1-phenylethanol had been converted, and then the conversion increases insignificantly for **4**, and even decreases for **3** (Table 3, Figure 5b). The amount of acetophenone obtained can be slightly increased from 65 to 72% when the amount of *t*-BuOOH oxidant was doubled with the other conditions being the same (Table 3, entries 23 and 26), while application of aq. 30% H_2_O_2_ or NaOCl as oxidants led to a sharp yield decrease to ca. 7 and 3%, respectively (Table 3, entries 28 and 29). This decrease in conversion may be due to the fast decomposition of the oxidants at high temperatures.

**Figure 6:**
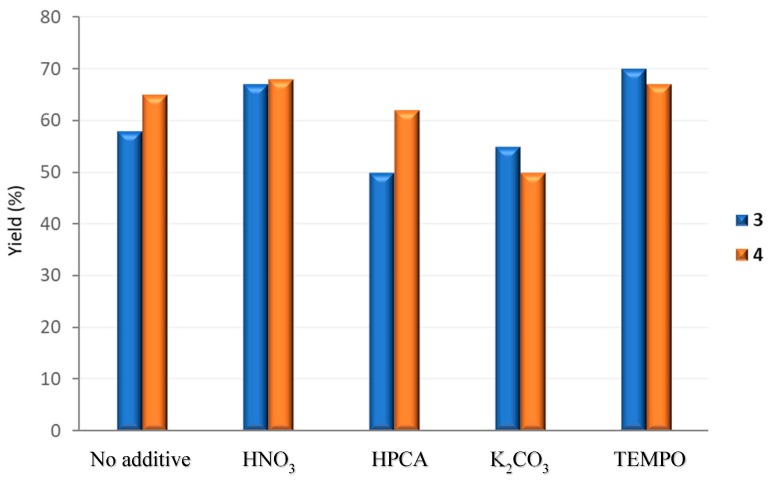
Influence of different additives on the yield of acetophenone. Reaction conditions: 5 mmol of substrate, 0.33 mmol of catalyst **3** or **4**, 5 mmol of *t*-BuOOH, 0.125 mmol of additive, 1 h, 80 °C, 5 W.

**Figure 7:**
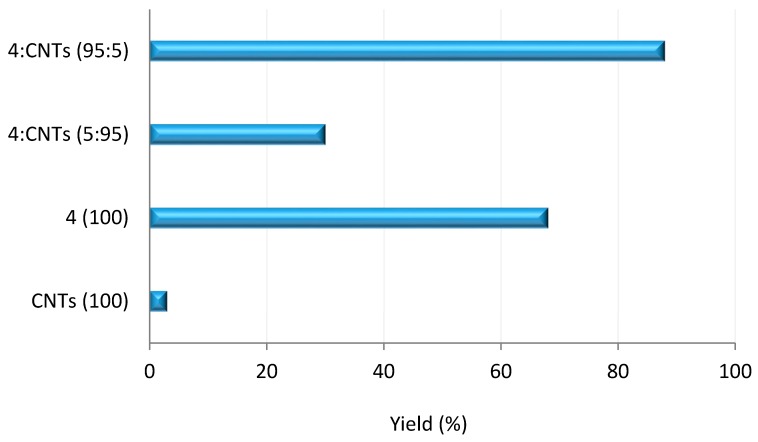
Influence of multiwalled carbon nanotube (MWCNTs) additive on the catalytic activity of **4**. Reaction conditions: 5 mmol of substrate, 0.33 mmol of catalyst, 5 mmol of *t*-BuOOH, 1 h, 80 °C, 5 W.

Several additives were also tested as possible promoters (Table 3). Thus, nitric (HNO_3_) [2,7,9,12,27] and pyrazinecarboxylic (Hpca) acids [27], 2,2,6,6-tetramethylpiperydil-1-oxyl (TEMPO) and K_2_CO_3_ [27,28,29,30] were chosen as the most promising additives (promoters) of the reaction. It was found that the additives have different effects, either of promotion or inhibition. Thus, the addition of nitric acid (0.125 mmol) has a small beneficial effect on both catalytic systems, resulting, for example, in a maximum yield of 67 and 68% for **3** and **4**, respectively (Table 3, entries 5 and 17, Figure 6). In contrast, Hpca has the opposite effect, i.e., a slight yield drop is observed (Table 3, entries 7 and 20). The radical TEMPO promoter caused a minor increase in the acetophenone yield for both catalytic systems, being more pronounced for the catalyst **3**. Since the TEMPO radical does not hamper the catalytic activity, the involvement of the free hydroxyl radical in the reaction mechanism is unlikely. The reaction can eventually involve a Fe(IV)=O species, also responsible for the high selectivity [31]. The promoting effect of TEMPO is known to occur in some copper-catalyzed alcohol oxidations where it acts as an H-abstractor from the metal coordinated alkoxide M-OCH(Me)Ph leading to the derived radical species M-OC^•^(Me)Ph and then to acetophenone Ph(Me)C=O upon an electron transfer [32]. The influence of carbon nanotubes (CNTs) on the catalytic activity of **4** also provides some promoting effect with a yield growth from 68 to 88% (Figure 7). 

#### 2.2.3. Effect of the Energy Input

**Figure 8:**
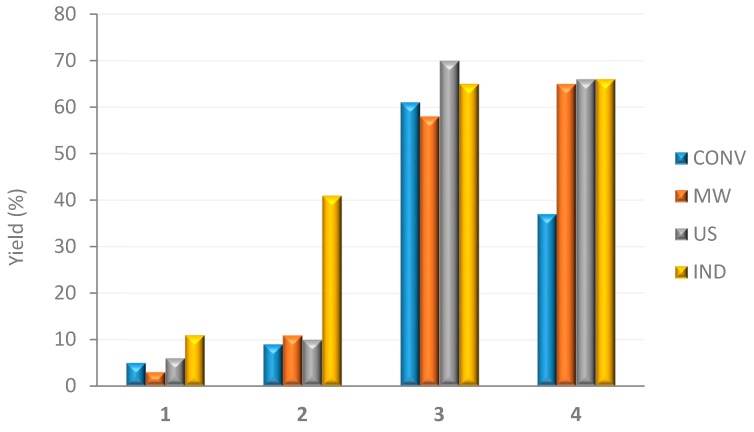
Influence of different energy-inputs on the yield of acetophenone. Reaction conditions: 5 mmol of substrate, 0.33 mmol of catalyst, 5 mmol of *t*-BuOOH, 1 h, 80 °C.

The effect of activating energy input on the catalytic output (Table 4, Figure 8) was controversial for different systems. Thus, the inductive heating increases the yield for catalyst **1** two-fold, while MW and US irradiations have no noticeable impact (Table 4, entries 1–4). The effect of inductive heating on the catalyst **2** is even more pronounced, i.e., yield raises more than four times from 9 to 41 %, whereas other types of activating irradiations do not influence much (Table 4, entries 6–10). The catalytic system **3** appears to be the one that preferably responds to ultrasound activation (Table 4, entries 11–15). Finally, the catalyst **4** responds equally to all three types of activating inputs (Table 4, entries 16–20). For comparative purpose, oxidation of 1-phenylethanol to acetophenone was also performed by mechanochemical treatment (500 rpm, 10 spheres of 10 mm diameter, with rotational inversions every 5 min) in the presence of **1**–**4**, at room temperature (Table 4, entries 5, 10, 15, and 20). Although the yields obtained by the ball-milling (BM) are modest (up to 17%), it should be noted that they were reached at room temperature, at which the other techniques yielded a residual amount of acetophenone. For now, it is difficult to draw any certain conclusion why the studied catalytic systems behave differently, and an additional thorough study will be performed in the future to address this point. 

The catalytic behavior of the materials **1** or **2** synthesized by wet or combined methods differ from those obtained by ball milling (**3** and **4**). As described above, in **1** and **2** there is the mixed Fe(II,III) oxide (Fe_3_O_4_), while **3** and **4** are composed of Fe^3+^ (originated from FeCl_3_ precursor) mixed with some Fe^2+^ from the FeSO_4_·(NH_4_)_2_·6H_2_O precursor. Fe_3_O_4_ is a metastable phase of iron oxide, which has an inverse spinel structure (space group Fd-3m) [33], and at the applied reaction conditions is ferrimagnetic [34], hence, it can agglomerate and respond to activation with a magnetic field of high frequency, in agreement with the obtained data (Table 4).

#### 2.2.4. Catalyst Recycling Studies

**Figure 9:**
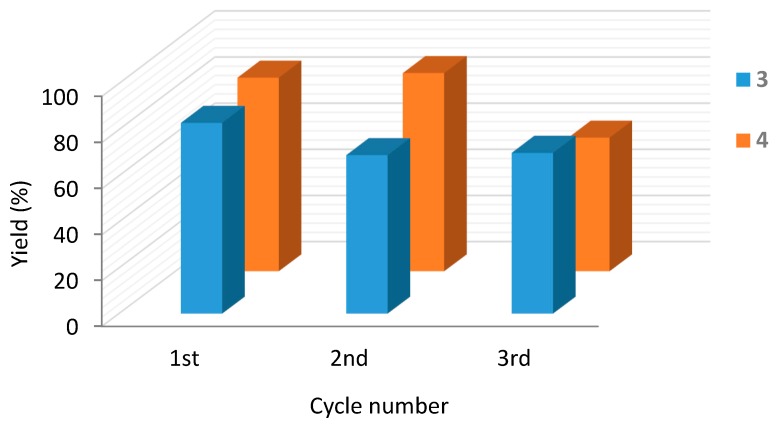
Recycling studies for **3** and **4**. Reaction conditions: 2.5 mmol of substrate, 2.5 mmol of *t*-BuOOH (aq. 70%), TEMPO additive (2.5 mol %), 80 °C, 3 h, microwave irradiation (5 W).

To check the possibility of reutilization of the most active catalysts **3** and **4**, we repeated the oxidation experiments after separation of the catalyst by centrifugation with subsequent washing with hexane and consecutive addition of new portions of substrate and oxidant. The iron composites become less efficient upon each new addition of 1-phenylethanol. However, after the third addition of the substrate (Table S3, Figure 9), the conversion of 1-phenylethanol is still observed to a good extent. 

## 3. Materials and Methods

### 3.1. Reagents and Instrumentation

Ammonium iron(II) sulfate hexahydrate ](NH_4_)_2_Fe(SO_4_)_2_·6H_2_O, (Merck, Darmstadt, Germany) anhydrous iron(III) chloride (FeCl_3_, Merck), iron(III) chloride hexahydrate (FeCl_3_⋅6H_2_O ≥ 98%, Sigma-Aldrich, Munich, Germany), sodium hydroxide (NaOH pellets, ≥98%, Sigma-Aldrich), DL-*sec*-phenethyl alcohol (Acros Organics, Geel, Belgium, 97%), *tert*-butyl hydroperoxide (*t*-BuOOH, 70% aq. solution, Acros Organics), acetonitrile (Ficher, Waltham, MA, USA) and benzaldehyde (Acros Organics) were used under normal ambient conditions, without additional purification. High purity MWCNTs (multiwalled carbon nanotubes) were kindly provided by Bayer Materials.

A planetary ball mill PM100/200 Retsch GmbH was equipped with a 250 mL grinding bowl, 10 stainless steel balls of 10 mm size and used for the ball-milling preparation of catalysts. FTIR spectra were obtained as diffuse reflectance (DRIFT) measurements on a Nicolet 6700 in the 400–4000 cm^−1^ with 2 cm^−1^ resolution. The powder diffuse reflectance spectra (UV-Vis-DRS, Shimadzu, Columbia, MD, USA) were recorded in the wavelength range of 200–800 nm using an ISR 2600 plus integration sphere in diffuse reflectance mode using BaSO_4_ as reference. Spectra were recorded at room temperature, and the data were transformed through the Kubelka–Munk function. Thermogravimetric analyses were carried out with a Perkin–Elmer instrument system (STA6000, Perkin-Elmer, MA, USA) at a heating rate of 10 °C·min^−1^ under a dinitrogen atmosphere, in the range from room temperature to ca. 800 °C. Diffraction (PXRD, Bruker, Madison, WI, USA) were collected in a D8 Advance Bruker AXS θ-2θ diffractometer, equipped with a LYNXEYE XE detector and a copper radiation source (Cu Kα, λ = 1.5406 Å), operated at 40 kV and 30 mA. 

Morphology and distribution of metal composites were characterized using a scanning electron microscope (SEM, FEGSEM and EDX) JEOL 7001F (JEOL, Akishima, Tokyo, Japan) with Oxford light elements EDX detector and EBSD detector X-ray photoelectron spectroscopy (XPS) characterization was performed using a dual anode XSAM800 spectrometer (KRATOS, Manchester, UK). Spectra were obtained using non-monochromatic Al Kα X-radiation (hν = 1486.6 eV) or Mg Kα X-radiation (hν = 1253.6 eV). Other operating conditions were described elsewhere [23]. For charge shift correction details, please see text. The sensitivity factors used for quantitative analyses were those of Vision2 for Windows, Version 2.2.9 (from KRATOS, Manchester, UK).

An Anton Paar Monowave 300 microwave reactor fitted with a rotational system and an IR temperature detector was used for the catalytic studies with a sealed cylindrical Pyrex reaction vessel (5 mL, 10 mm internal diameter). The ultrasonic experiments were performed using an ATU ultrasonic thermoregulated bath. For inductive heating, a commercial portable induction hob (model: MX-PIP2195) has been used. Ferromagnetic flat plate (140 × 140 × 1 mm) with square shape and round hole Ø 25 cm in the center has been placed on top of the hob to initiate it. The reactor has been positioned in the center of the hole with stirring from above. GC analysis was undertaken by using a Fisons Instruments GC 8000 series gas chromatograph with a DB-624 (J&W) capillary column (DB-WAX, column length: 30 m; internal diameter: 0.32 mm), FID detector, and the Jasco–Borwin v.1.50 software. The temperature of injection was 240 °C. The initial temperature was maintained at 120 °C for 1 min, then raised from 10 °C·min^−1^ to 200 °C and held at this temperature for 1 min. Helium was used as the carrier gas.

### 3.2. Catalyst Preparation

For comparison purposes, the preparation of catalysts (Figures S1–S4) was carried out by several (dry, wet and combined) methods as follows.

**1**: In 100 mL round bottom flask, 0.7843 g, 2 mmol of (NH_4_)_2_Fe(SO_4_)_2_·6H_2_O and 1.0812 g, 4 mmol of FeCl_3_·6H_2_O were added to 80 mL of deionized water and the solution was magnetically stirred (600 rpm) at 50 °C. Total of 1 M NaOH was added dropwise to the solution until pH 10–12. Then the reaction was stopped, the formed precipitate was washed with water and ethanol and dried overnight at 80 °C.

**2**: Total of 0.39 g (1 mmol) (NH_4_)_2_Fe(SO_4_)_2_·6H_2_O and 0.54 g (2 mmol) FeCl_3_·6H_2_O were treated in ball mill (500 rpm, 10 spheres of 10 mm diameter) at room temperature for 10 min, with rotational inversions every 5 min. The thus obtained orange solid was transferred to a glass vial and 2 mL of deionized water were added. Then 1 M NaOH was added until pH 10–12 and the suspension was stirred for 30 min. After that, the formed black precipitate was filtered off, washed with water and ethanol, and dried overnight at 80 °C.

**3**: Total of 3.14 g (8 mmol) (NH_4_)_2_Fe(SO_4_)_2_·6H_2_O) and 2.61 g (16 mmol) FeCl_3_ were mixed and ball milled (500 rpm, 10 spheres of 10 mm diameter) at room temperature for 30 min, with rotational inversions every 5 min. The thus formed reddish-brown powder was dried overnight at 80 °C.

**4**: Total of 0.25 g (6.2 mmol) NaOH, 3.32 g (8 mmol) (NH_4_)_2_Fe(SO_4_)_2_·6H_2_O) and 2.61 g (16 mmol) FeCl_3_ were treated in ball mill as indicated above. The formed brown powder was dried overnight at 80 °C.

### 3.3. Catalytic Studies

The microwave-assisted oxidations were performed in a cylindric Pyrex tube to which 5 mmol 1-phenylethanol, 50–500 μmol catalyst and 5 mmol of 70% aq. solution of *t*-BuOOH were added in this order. The tube was then sealed and kept for 1 h at 80 °C under microwave irradiation (5–10 W). Finally, 5 mL of MeCN (to extract substrate and products from the reaction mixture) and 300 µL of benzaldehyde (internal standard) were added. The obtained mixture was stirred for 5 min and then a sample (1 µL) was taken from the organic phase and analyzed by GC using benzaldehyde as an internal standard method.

Under the optimized conditions, several energy inputs were tested. A total of 2.5 mmol 1-phenylethanol, 0.33 mmol catalyst, and 2.5 mmol of 70% aq. solution of *t*-BuOOH were added to a 10 mL Pyrex reactor and the reaction proceeded under US irradiation at 80 °C for 1h. Induction heating was performed as follows: the reaction mixture was added to a 10 mL glass vial as indicated above and the vial was placed on the center of the induction hob covered with a steel plate with a hole. The device was turned on with an induction power of 200 W for 1 h. The temperature was measured at the end of the reaction being ca. 70 °C. For reactions under conventional heating, a 10 mL glass reactor with the reaction mixture analogous to the ones described above was placed into an oil bath and heated for 1 h at 80 °C. At the end the product was extracted with MeCN and analyzed by GC with benzaldehyde used as an internal standard.

## 4. Conclusions

Wet, dry, and combined synthetic methods were used to prepare iron materials with different compositions, particle sizes (0.1–1 µm), shapes (semi-spherical, needles, and rectangles), and magnetic susceptibilities (paramagnetic or diamagnetic). Moreover, the materials prepared by wet, dry, and combined methods from the same starting materials (namely (NH_4_)_2_Fe(SO_4_)_2_·6H_2_O and FeCl_3_·6H_2_O) catalytically behave differently; the dry mechanochemical preparation, can increase the catalytic activity more than six-fold. It is also demonstrated that the activation of catalysts by ultrasounds and an oscillating magnetic field of high frequency can significantly increase the overall effectiveness of a catalytic system. Finally, the prepared catalysts can be magnetically recovered and reused at least three times.

## Figures and Tables

**Table 1:** XPS quantifications of **1**–**4**.

	1	2	3	4		
	Atomic concentrations (%)					
C	11.4	19.5	45.7	52.2		
O	43.8	59.3	18.5	21.2		
Cl	2.4	0.8	20.6	11.6		
N	0.0	1.3	6.7	6.4		
S	0.0	0.0*	1.2	1.6	* traces	
Fe	26.3	19.3**	7.4	6.8	** underestimated (see text)	
Na	16.1	0.0	—	0.0		
					Atomic ratios in precursors or oxides	
	Experimental atomic ratios				(NH_4_)_2_Fe(SO_4_)_2_·6H_2_O	FeCl_3_ (6H_2_O)
N/Fe		0.06	0.9	0.9	2.0	
S/Fe			0.2	0.2	2.0	
Cl/Fe	0.09	0.04	2.8	1.7		3.0
					Fe_3_O_4_	
O_530 eV_/Fe	1.3	1.7	***	***	1.3	*** O_530 eV_ in the tail of O 1s

* traces: not quantified residual amount; ** underestimated (see text); *** O_530 eV_ in the tail of O 1s: oxygen bonded to iron (expected at ~530 eV), is a unresolved component, included in the O 1s tail at the low BE side, therefore the ratio O_530 eV_/Fe was not computed for samples **3** and **4**.

**Table 2:** MW-assisted oxidation of 1-phenylethanol to acetophenone. ^a^

Entry	Catalyst	Catalyst Amount (mmol)	Yield (%) ^b^	TOF ^c^
1	-	-	<1	-
2	FeCl_3_·6H_2_O	1	28	1
3	FeCl_3_ anhydrous	1	11	<1
4	FeSO_4_·(NH_4_)_2_·6H_2_O	1	2.5	<1
5	**1**	0.05	2.3	2
6		0.1	2.2	1
7		0.33	2.7	<1
8		0.5	3.3	<1
9	**2**	0.05	3.8	4
10		0.1	5	3
11		0.33	7.6	1
12		0.5	11	1
13	**3**	0.05	25	25
14		0.1	52	26
15		0.33	58	9
16		0.5	61	6
17	**4**	0.05	19	19
18		0.1	39	20
19		0.33	65	10
20		0.5	65	7

^a^ Reaction conditions: 5 mmol substrate, 5 mmol *t*-BuOOH (aq. 70%), 0.05–0.5 mmol Fe catalyst, 80 °C, 1 h, microwave irradiation (5–10 W). ^b^ Molar yield (%) based on substrate, i.e., moles of product per 100 mol of substrate determined by GC. ^c^ TOF (turnover frequency) = TON (turnover number) per hour; TON = number of moles of product per mol of catalyst.

**Table 3:** MW-assisted solvent-free oxidation of 1-phenylethanol to acetophenone by **3** and **4**. ^a^

Entry	Catalyst	Reaction Time (h)	Temperature (°C)	Additive (mmol)	Yield (%) ^b^	TOF ^c^
1	**3**	0.5	80	-	52	16
2		1	80	-	58	9
3		1.5	80	-	72	7
4		3	80	-	69	3
5		1	80	HNO_3_ (0.125)	67	10
6		1	80	K_2_CO_3_ (0.125)	55	8
7		1	80	HPCA (0.125)	50	8
8		1	80	TEMPO (0.125)	70	11
9		1	60	-	36	5
10		1	80	-	58	9
11		1	100	-	70	11
12		1	120	-	61	9
13	**4**	0.5	80	-	55	17
14		1	80	-	65	10
15		1.5	80	-	73	11
16		3	80	-	82	12
17		1	80	HNO_3_ (0.125)	68	10
18		1	80	K_2_CO_3_ (0.125)	50	8
19 ^d^		1	80	K_2_CO_3_ (0.125)	19	3
20		1	80	HPCA (0.125)	62	9
21		1	80	TEMPO (0.125)	67	10
22		1	60	-	18	3
23		1	80	-	65	10
24		1	100	-	68	10
25		1	120	-	67	10
26 ^e^		1	80	-	72	11
27 ^f^		1	80	-	2.5	<1
28 ^g^		1	80	-	6.7	1
29 ^h^		1	80	-	2.9	<1

^a^ Reaction conditions: 5 mmol substrate, 5 mmol aq. 70% *t*-BuOOH (substrate/*t*-BuOOH molar ratio = 1:1), 0.33 mmol **3** or **4**, 60–120 °C, 0.3–3 h, MW irradiation (5–25 W). ^b^ Molar yield (%) based on substrate, i.e., moles of product per 100 mol of substrate determined by GC. ^c^ TOF = TON per hour; TON = number of moles of product per mol of catalyst. ^d^ 1 M aqueous solution of K_2_CO_3_. ^e^ Substrate/*t*-BuOOH molar ratio = 1:2. ^f^ Under air and without other oxidants apart from dioxygen from atmosphere. ^g^ Using H_2_O_2_ 30% aq. solution as an oxidant (1:1). ^h^ Using NaOCl as an oxidant (1:1).

**Table 4:** Effect of the different energy inputs for the oxidation of 1-phenylethanol to acetophenone by **1**–**4**. ^a^

Entry	Catalyst	Method ^b^	Temperature (°C)	Yield (%) ^c^	TOF ^d^
1	**1**	CONV	80	5	1
2		MW	80	3	<1
3		US	80	6	1
4		IND	70	11	2
5		BM	r.t.	5	1
6	**2**	CONV	80	9	1
7		MW	80	11	2
8		US	80	10	2
9		IND	70	41	6
10		BM	r.t.	7	1
11	**3**	CONV	80	61	9
12		MW	80	58	9
13		US	80	70	11
14		IND	70	65	10
15		BM	r.t.	2	<1
16	**4**	CONV	80	37	6
17		MW	80	65	10
18		US	80	66	10
19		IND	70	66	10
20		BM	r.t.	17	4

^a^ Reaction conditions: 5 mmol substrate, 5 mmol *t*-BuOOH (aq. 70%), 0.33 mmol Fe composite, 1 h. ^b^ CONV = under conventional heating with oil bath; MW = under MW irradiation (5 W); US = under ultrasound irradiation; IND = under induction heating, BM = ball milling (500 rpm, 10 spheres of 10 mm diameter, room temperature, with rotational inversions every 5 min).^c^ Molar yield (%) based on substrate, i.e., moles of product per 100 mol of substrate determined by GC. ^d^ TOF = TON per hour; TON = number of moles of product per mol of catalyst.

## Supplementary Materials

The following are available online. Preparation details, FTIR, and UV-vis spectra, thermogravimetric analysis data, X-ray diffraction patterns, magnetic susceptibilities, and data on influence of various factors on the catalytic activity.
