# Supplementary material for: Ultrasound and Radiation-Induced Catalytic Oxidation of 1-Phenylethanol to Acetophenone with Iron-Containing Particulate Catalysts
Source: Molecules. 2020 Feb 8;25(3):740. doi: 10.3390/molecules25030740 (PMC7038031; doi:10.3390/molecules25030740)
Supplement: Supplementary file 1 [file molecules-25-00740-s001.pdf]

Supplementary Materials

Ultrasound and Radiation-Induced Catalytic Oxidation of 1-Phenylethanol to Acetophenone with Iron-Containing Particulate Catalysts

Mohamed M. A. Soliman [1,2], Maximilian N. Kopylovich [1,*], Elisabete C. B. A. Alegria [1,3,*], Ana P. C. Ribeiro [1,*], Ana M. Ferraria [4], Ana M. Botelho do Rego [4], Luís M. M. Correia [3], Marta S. Saraiva [2,5] and Armando J. L. Pombeiro [1]

1 Centro de Química Estrutural, Complexo I, Instituto Superior Técnico, Universidade de Lisboa, 1049-001 Lisboa, Portugal; mohamed.soliman@tecnico.ulisboa.pt (M.M.A.S.); pombeiro@tecnico.ulisboa.pt (A.J.L.P.)

2 Centro de Química e Bioquímica, Departamento de Química e Bioquímica, Faculdade de Ciências, Universidade de Lisboa, 1749-016 Lisboa, Portugal; mssaraiva@fc.ul.pt

3 Área Departamental de Engenharia Química, ISEL, Instituto Politécnico de Lisboa, 1959-007 Lisboa, Portugal; luiscorreia1994@gmail.com

4 BSIRG, IBB-Institute for Bioengineering and Biosciences, Departamento de Engenharia Química, Instituto Superior Técnico, Universidade de Lisboa, 1049-001 Lisboa, Portugal; ana.ferraria@tecnico.ulisboa.pt (A.M.F.); amrego@tecnico.ulisboa.pt (A.M.B.d.R.)

5 BioISI -Biosystems & Integrative Sciences Institute, Departamento de Química e Bioquímica, Faculdade de Ciências, Universidade de Lisboa, Campo Grande, 1049-001 Lisboa, Portugal

* Correspondence: maximilian.kopylovich@tecnico.ulisboa.pt (M.N.K.); ebastos@deq.isel.ipl.pt (E.C.B.A.A.); apribeiro@tecnico.ulisboa.pt (A.P.C.R.); Tel.: +351-218317163 (E.C.B.A.A.)

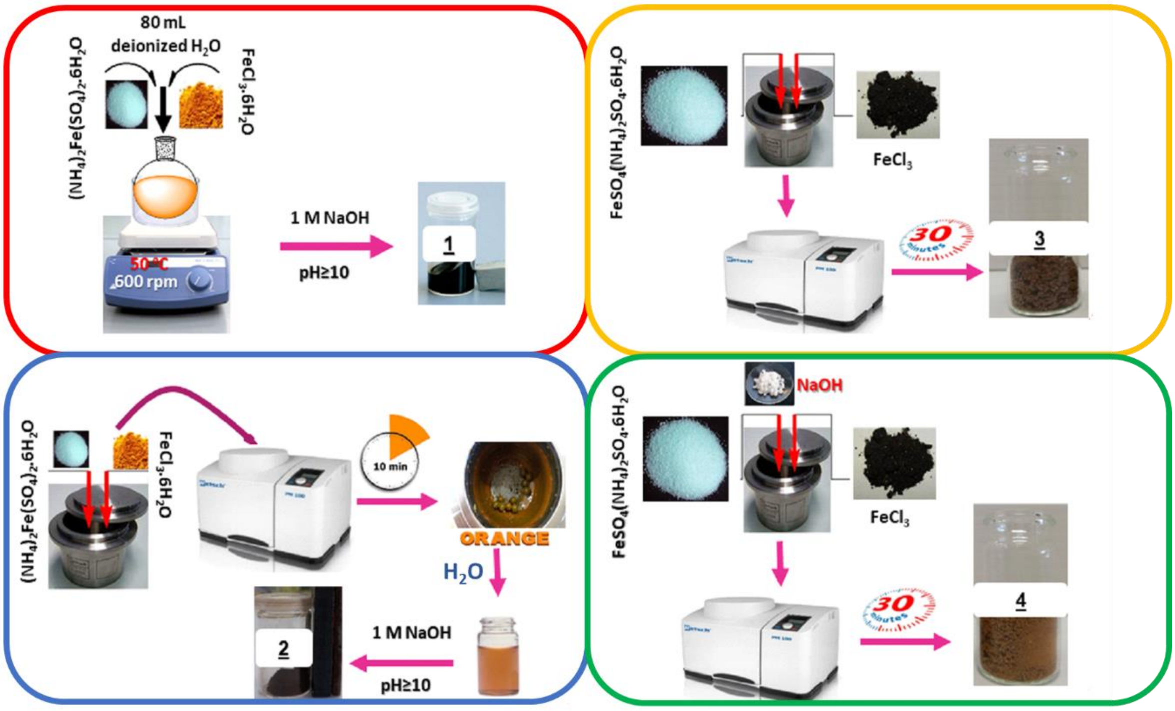

Figure S1. Preparation methods of compounds **1–4**.

Table S1. Details on the preparation of **1–4**.

**1**	**2**	**3**	**4**
2 mmol	1 mmol	8 mmol	8 mmol
$(NH_4)_2Fe(SO_4)_2{\cdot}6H_2O$	$(NH_4)_2Fe(SO_4)_2{\cdot}6H_2O$	$(NH_4)_2Fe(SO_4)_2{\cdot}6H_2O$	$(NH_4)_2Fe(SO_4)_2{\cdot}6H_2O$
4 mmol	2 mmol	16 mmol	16 mmol
$FeCl_3{\cdot}6H_2O$	$FeCl_3{\cdot}6H_2O$	$FeCl_3$	$FeCl_3$
DIW	BM	BM	BM
50 ˚C	500 rpm, 10 sph, 10 min, RT	500 rpm, 10 sph, 30 min, RT	500 rpm, 10 sph, 30 min, RT
NaOH [1 M]	NaOH [1 M]	Without NaOH	NaOH [6 mmol]
Wet	Mixed	Dried	Dried

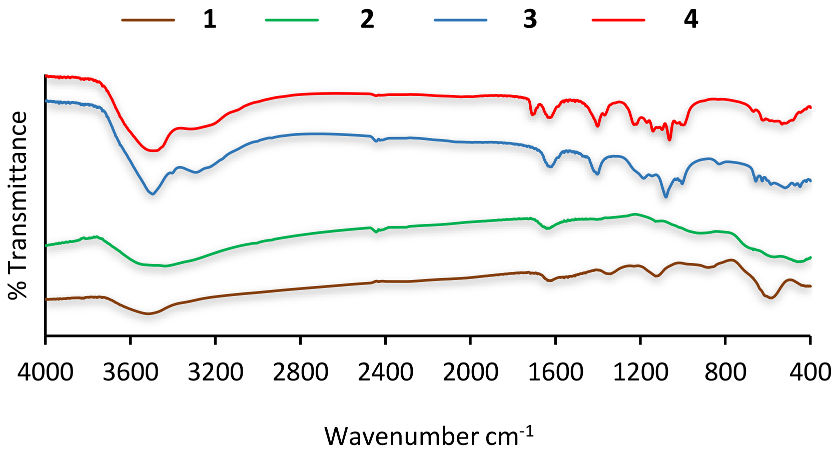

Figure S2. FTIR spectra of **1–4** in the range 4000–400 cm^{-1}.

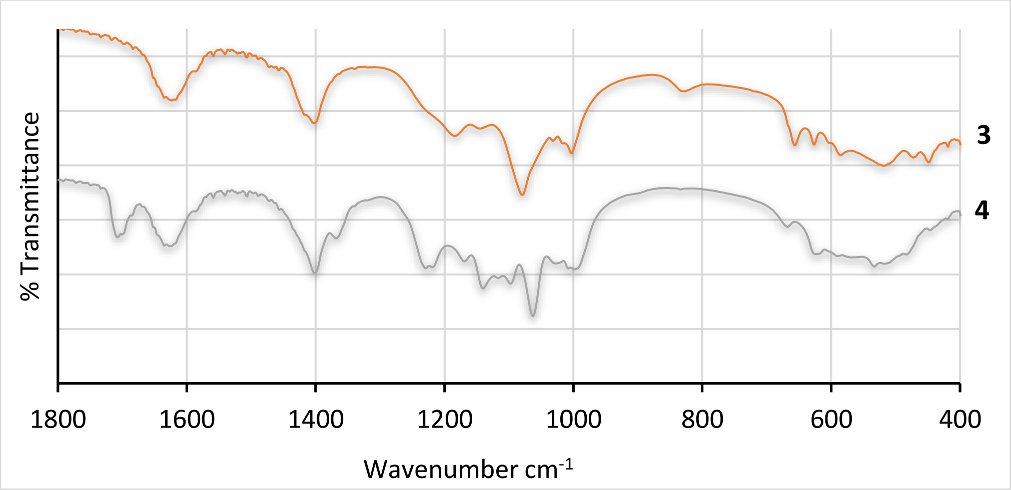

Figure S3. FTIR spectra of **3** and **4** in the range 1800–400 cm^{-1}.

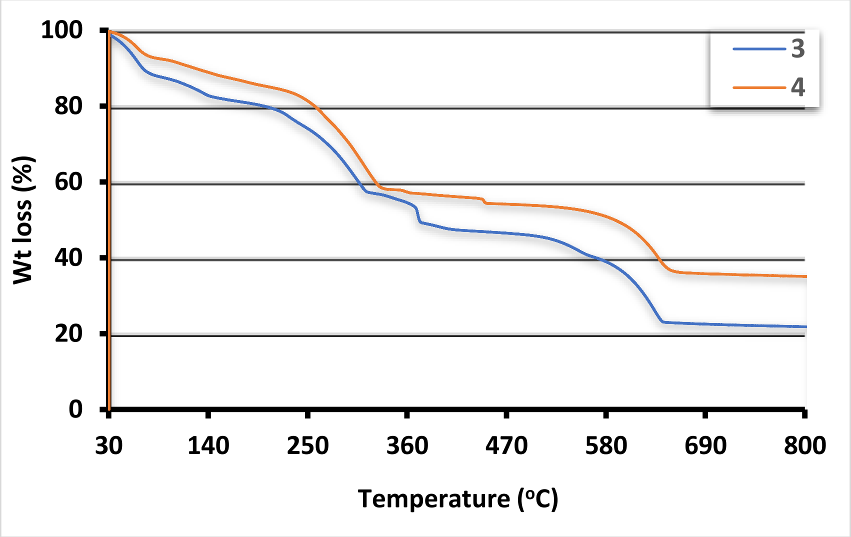

Figure S4. Thermogravimetric analysis of **3** and **4**

(a)

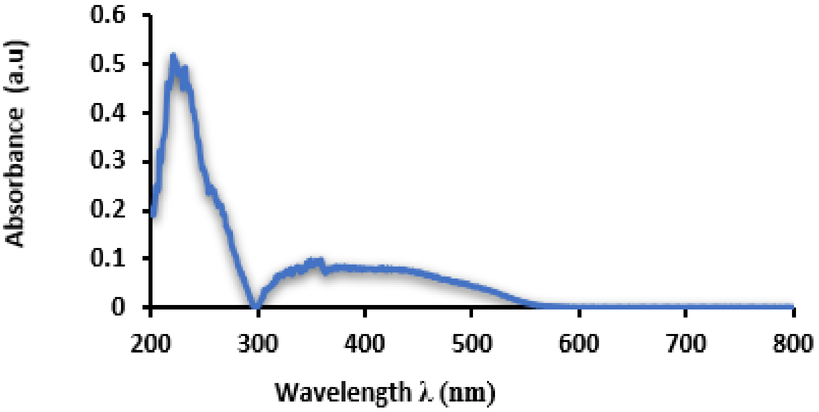

Absorbance (a.u)
0.6
0.5
0.4
0.3
0.2
0.1
0
200
300
400
500
600
700
800
Wavelength λ (nm)

(b)

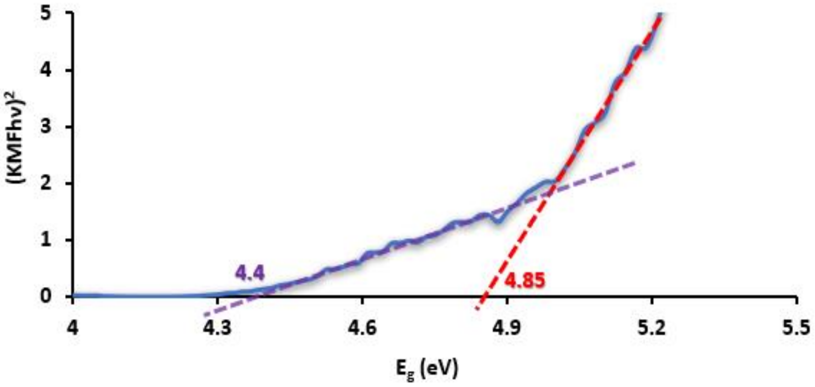

(KMFhν)²
5
4
3
2
1
0
4.4
4.85
4
4.3
4.6
4.9
5.2
5.5
Eg (eV)

(c)

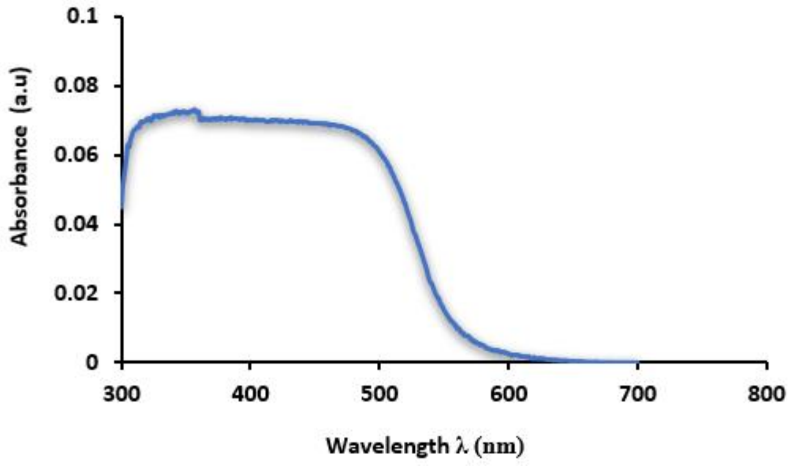

Absorbance (a.u)
0.1
0.08
0.06
0.04
0.02
0
300
400
500
600
700
800
Wavelength λ (nm)

(d)

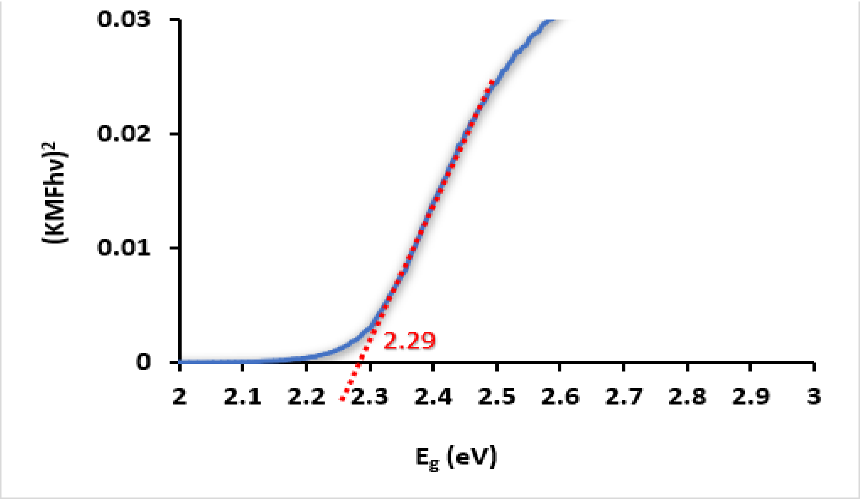

Figure S5. Diffuse reflectance absorbance spectra of **3** (a,b) and **4** (c,d).

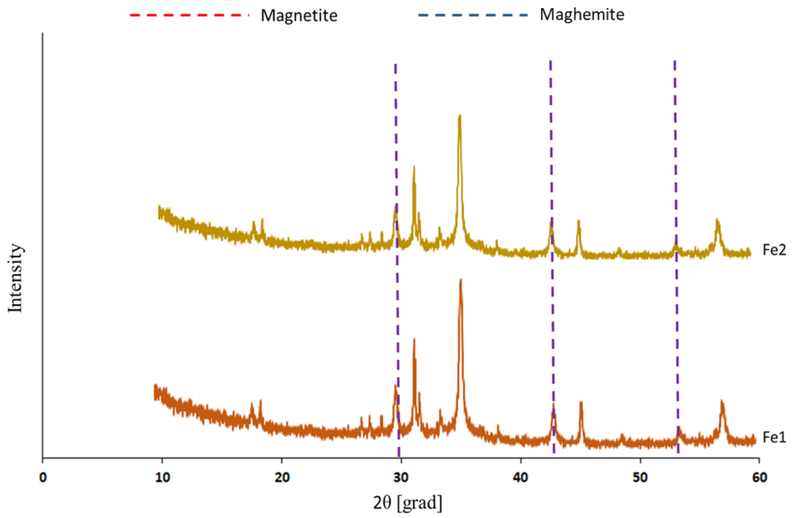

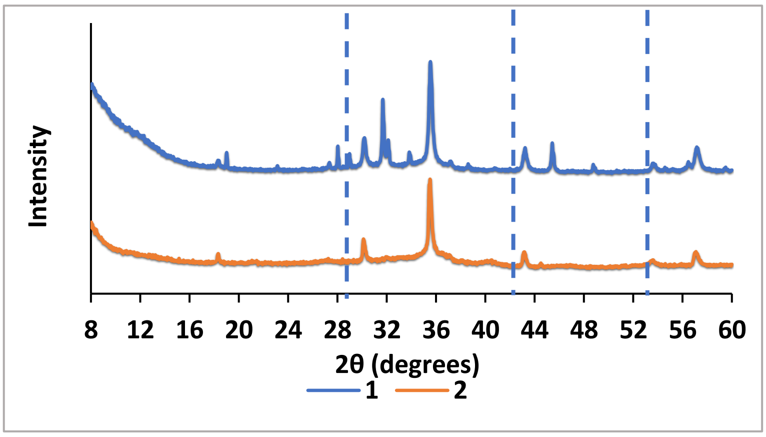

Figure S6. X-ray diffraction patterns of **1** and **2**. Red for magnetite (PDF Card#04- 015-3100); blue for maghemite (PDF Card#00-039-1346)

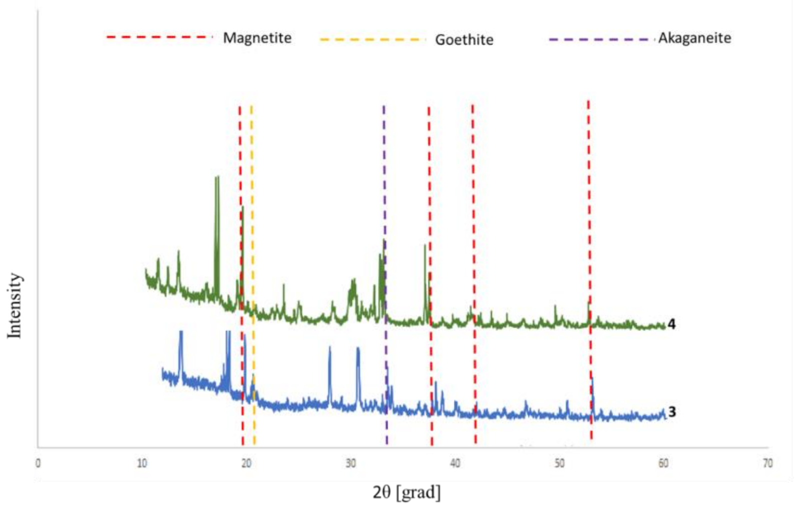

Figure S7. X-ray diffraction patterns of **3** and **4**. Red for magnetite (PDF Card#04- 015-3100); purple for akaganeite (PDF Card# 00-39-1346) and yellow for goethite (PDF Card# 00-34-1266)

Table S2. Magnetic susceptibility of the studied materials.

Material	Mass susceptibility X_g (c.g.s)	Molar Susceptibility[a], X_m	
**1**	0.179×10^{-3}	0.0283	Paramagnetic
**2**	0.314×10^{-3}	0.0506	Paramagnetic
**4**	-1.449×10^{-5}	−0.00345	Diamagnetic

[a] The molar susceptibility, χ_m, is calculated by multiplying X_g by the molecular mass of the substance. The molar susceptibility is positive if the substance is paramagnetic and negative if the substance is diamagnetic.
Note: For the catalyst **3**, it was impossible to measure the magnetic susceptibility since it is highly hydroscopic upon exposure to air.

Table S3. Recycling of **3** and **4** in MW-assisted solvent-free oxidation of 1-phenylethanol to acetophenone.

Cycle	Yield (%)[b]
**3**	
1st	83
2nd	69
3rd	70
**4**	
1st	84
2nd	86
3rd	58

Reaction conditions: 2.5 mmol of substrate, 2.5 mmol of t-BuOOH (aq. 70%), TEMPO additive (2.5 mol %), 80 ºC, 3 h, microwave irradiation (5 W).
